# Evaluating the effect of the Helping Mothers Survive Bleeding after Birth (HMS BAB) training in Tanzania and Uganda: study protocol for a randomised controlled trial

**DOI:** 10.1186/s13063-017-2056-7

**Published:** 2017-07-06

**Authors:** Claudia Hanson, Andrea B. Pembe, Fadhlun Alwy, Susan Atuhairwe, Sebalda Leshabari, Jessica Morris, Frank Kaharuza, Gaetano Marrone, the HMS BAB study team

**Affiliations:** 1 Department of Public Health Sciences, Karolinska Institutet, Solna, Sweden; 2 Department of Disease Control, London School of Hygiene and Tropical Medicine, London, UK; 3 Department of Obstetrics and Gynaecology, Muhimbili University of Health and Allied Sciences, Dar es Salaam, Tanzania; 4 Association of Gynaecologists and Obstetricians of Tanzania (AGOTA), Dar es Salaam, Tanzania; 5 Association of Obstetricians and Gynaecologists of Uganda (AOGU), Kampala, Uganda; 6 Tanzania Midwives Association (TAMA), Dar es Salaam, Tanzania; 7 International Federation of Gynaecology and Obstetrics (FIGO), London, UK; 8 Makerere University School of Public Health, Kampala, Uganda

**Keywords:** Postpartum haemorrhage, Competency-based training, Simulation training, Helping Mothers Survive Bleeding after Birth, Cluster-randomised trial

## Abstract

**Background:**

Postpartum haemorrhage complicates approximately 10% of all deliveries and contributes to at least a quarter of all maternal deaths worldwide. The competency-based Helping Mothers Survive Bleeding after Birth (HMS BAB) training was developed to support evidence-based management of postpartum haemorrhage. This one-day training includes low-cost MamaNatalie® birthing simulators and addresses both prevention and first-line treatment of haemorrhage. While evidence is accumulating that the training improves health provider’s knowledge, skills and confidence, evidence is missing as to whether this translates into improved practices and reduced maternal morbidity and mortality. This cluster-randomised trial aims to assess whether this training package — involving a one-day competency-based HMS BAB in-facility training provided by certified trainers followed by 8 weeks of in-service peer-based practice — has an effect on the occurrence of haemorrhage-related morbidity and mortality.

**Methods/design:**

In Tanzania and Uganda we randomise 20 and 18 districts (clusters) respectively, with half receiving the training intervention. We use unblinded matched-pair randomisation to balance district health system characteristics and the main outcome, which is in-facility severe morbidity due to haemorrhage defined by the World Health Organizationation-promoted disease and management-based near-miss criteria. Data are collected continuously in the intervention and comparison districts throughout the 6-month baseline and the 9-month intervention phase, which commences after the training intervention. Trained facility midwives or clinicians review severe maternal complications to identify near misses on a daily basis. They abstract the case information from case notes and enter it onto programmed tablets where it is uploaded.

Intention-to-treat analysis will be used, taking the matched design into consideration using paired *t* test statistics to compare the outcomes between the intervention and comparison districts. We also assess the impact pathway from the effects of the training on the health provider’s skills, care and interventions and health system readiness.

**Discussion:**

This trial aims to generate evidence on the effect and limitations of this well-designed training package supported by birthing simulations. While the lack of blinding of participants and data collectors provides an inevitable limitation of this trial, the additional evaluation along the pathway of implementation will provide solid evidence on the effects of this HMS BAB training package.

**Trial registration:**

Pan African Clinical Trials Registry, PACTR201604001582128. Registered on 12 April 2016.

**Electronic supplementary material:**

The online version of this article (doi:10.1186/s13063-017-2056-7) contains supplementary material, which is available to authorized users.

## Background

Without intensifying efforts to improve the quality of intrapartum care, many mothers will continue to die while giving life, and the renewed promise of the Sustainable Development Goals will not be achieved [1]. It is estimated that 201,000 women died in pregnancy and childbirth in sub-Saharan Africa alone in 2015 [2]. Postpartum haemorrhage (PPH) is the most common cause of maternal mortality; 29% of maternal deaths are attributable to this complication [3]. Between 11% and 17% of women who give birth in eastern Africa experience PPH [4, 5] with at least one in 1000 women dying due to haemorrhage and 30 more suffering a severe morbidity as a result [6, 7].

Capacity building of providers to prevent, identify and treat complications is one critical element of health system strengthening. It has been increasingly recognised that training must be tailored specifically to the needs of the health workers and their work conditions [8]. Recent evidence has also demonstrated that to build capacity, competency-based training may bring the best results in terms of improving health care providers’ skills and knowledge [9]. Competency-based learning is an approach that defines individual skills or learning outcomes as a competency and defines small units of learning. Learners work on one competency at a time, which is likely to be a small component of a larger learning goal. This approach is distinct from broader courses which were historically preferred. Often such competency-based training packages also employ simulation methods to improve skills and confidence.

Several competency-based training packages are currently employed in low-resource settings. One example is the *Helping Babies Breathe* training package, which addresses neonatal care. Evaluations of this package have shown a positive effect on providers’ competence and on neonatal mortality and stillbirth rates [10–12], though not in all settings [13, 14]. Another example is the *PRONTO* (*Programa de Rescate Obstétrico y Neonatal: Tratameniento Óptimo Y Oportuno*) competency-based training package. This package showed an effect on reducing neonatal mortality in Mexico [15] and resulted in improved use of evidence-based interventions, patient-centred care and teamwork in Guatemala [16]. The *Making it Happen* programme, a comprehensive simulation and competency-based emergency obstetric care training, was very well received by health providers and demonstrated improvements in knowledge and competence of providers [17, 18].

The *Helping Mothers Survive Bleeding after Birth* (HMS BAB) training programme is a one-day competency training specifically addressing basic skills to prevent and treat PPH [19, 20]. The training is conceptualised to build the capacity of teams of obstetricians, midwives and other health care providers attending births. Recent studies have shown this HMS BAB training to have an impact on knowledge and confidence among health care providers [21, 22]. While evidence on the effect on health outcomes of competency-based training, especially the Helping Babies Breathe training, is accumulating, there is no evidence of the effect of the HMS BAB training on health outcomes. It is also unknown whether health system challenges, such as lack of equipment and supplies, counteract the effectiveness of such a training programme.

This study evaluates the effect of the HMS BAB one-day competency-based in-facility training followed by 8 weeks of in-service practice along the impact pathway, including the health outcomes of morbidity and mortality in the two low-resource settings of Tanzania and Uganda. We will specifically assess the effect of the training on (1) severe maternal morbidity and mortality due to PPH, (2) preventive actions and case management of PPH, (3) knowledge, skills and confidence in preventing and managing PPH and (4) facility readiness, including equipment and supplies, for prevention and care of PPH.

The study is implemented as a joint project by professional associations of gynecologists and obstetricians and midwives in both countries with the support of the International Federation of Gynaecology and Obstetrics (FIGO) and the International Confederation of Midwives (ICM) with the sub-aim to foster within-country collaborations of the associations.

## Methods/design

### Study design and methodology

**Fig. 1:**
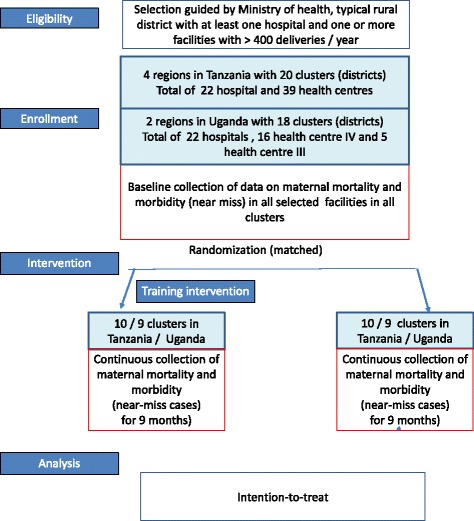
Trial design

**Fig. 2:**
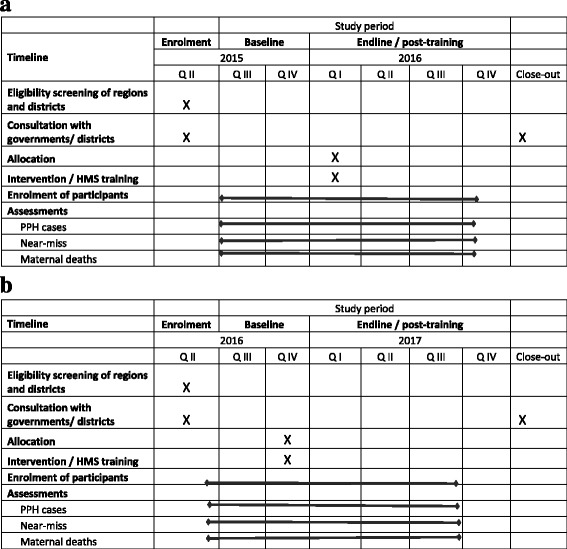
**a** HMS BAB enrolment and follow-up in Tanzania. (baseline data collection is running from July to December 2015 and endline / post training from January to October 2016, the HMS BAB intervention took place in January 2016). **b** HMS BAB enrolment and follow-up in Uganda. (baseline data collection is running from June to November 2016 and endline / post training from December 2016 to September 2017, the HMS BAB intervention took place in December 2017)

The study uses a cluster-randomised design (the Standard Protocol Items: Recommendations for Interventional Trials (SPIRIT) checklist is provided as Additional file 1). We compare ten districts in Tanzania and nine districts in Uganda where the HMS BAB training is being implemented in all hospitals and selected high case-load referral health centres with ten and nine comparison districts in each country respectively that do not receive this training (Fig. 1). All selected facilities undergo a 6-month baseline assessment of the outcomes after which the training intervention is implemented (Fig. 2). All districts, both intervention and comparison, are monitored for a further 9-month period. After this period the facilities in the comparison districts receive the training intervention.

### Study area

**Fig. 3:**
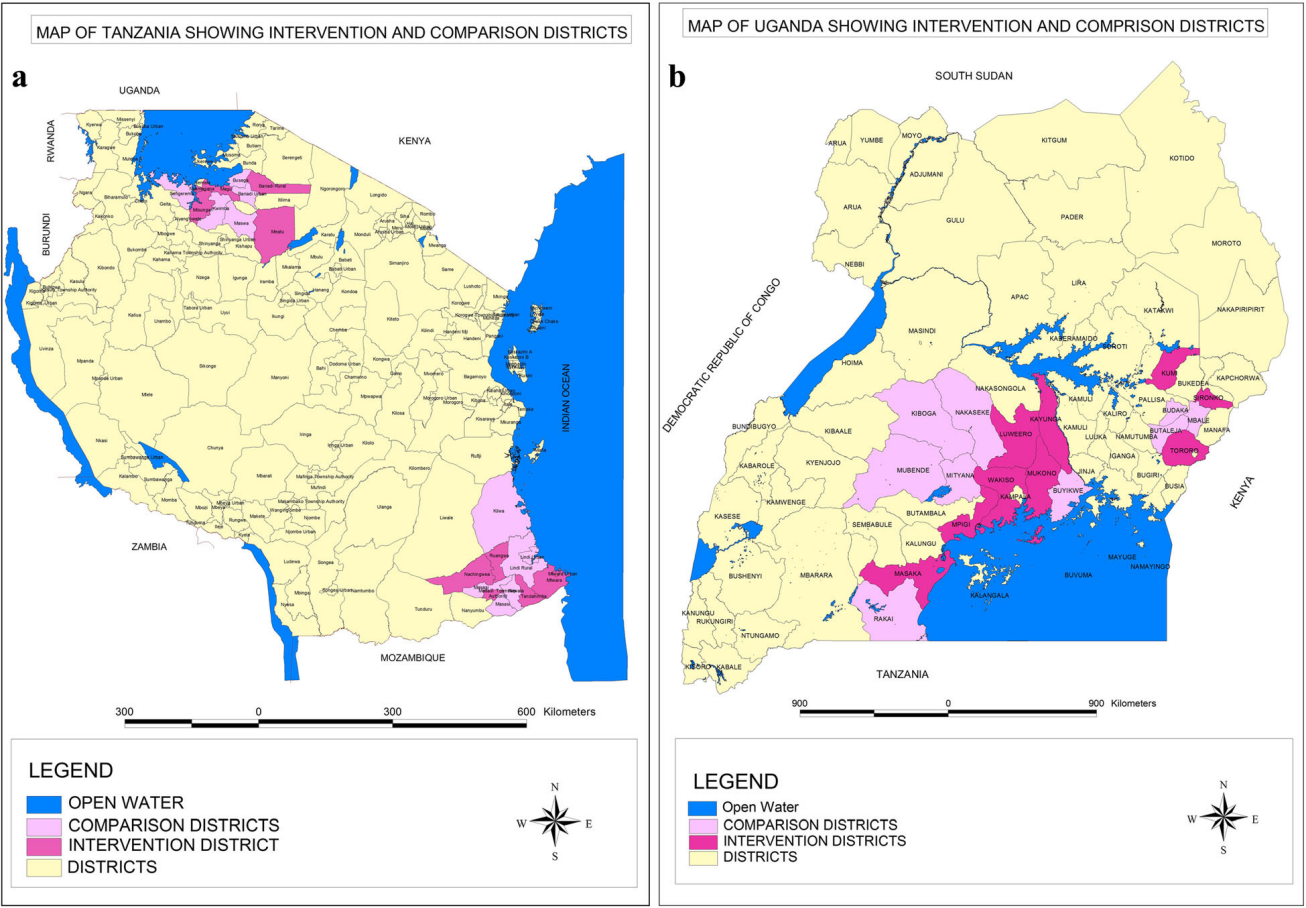
Maps of the intervention and comparison districts for **a** Tanzania and **b** Uganda

In Tanzania, the study is being implemented in ten primarily rural districts in southern Tanzania (Mtwara and Lindi regions) and ten districts in north-eastern Tanzania (Mwanza and Simiyu regions) (Fig. 3). The study areas include small urban regional and district capitals. Mwanza region includes a larger urban district of Mwanza town with approximately half a million people. Both settings have high maternal mortality ratios [23, 24]. Numerous reports have been published on the limited readiness of the health facilities in these regions to deal with maternal complications and the insufficient skills and practices of the health workers [25–27].

In Uganda, this study is being implemented in seven districts in the Eastern region and 11 districts in the Central region. Maternal mortality is estimated to be 343 deaths per 100,000 live births in Uganda [2]. Morbidity due to complications in pregnancy and childbirth has been described as high in the Central region [28]. Similar to Tanzania, health system supply issues and lack of competencies and knowledge limit the effective use of measures to prevent and treat PPH [29].

### The intervention

The HMS BAB training is a competency-based training package developed by Jhpiego and Laerdal Global Health. The training programme has been designed to improve the knowledge and performance of delivery care providers through (1) an educational environment at the health facility level, with training conducted in health facilities; (2) an improved clinical practice environment, with material for practicing simulations provided in labour rooms; (3) monitoring quality improvement through ongoing data collection and feed-back analysis reports [30].

The training is provided by Jhpiego-accredited HMS BAB master trainers and is conceptualised to be relevant to all cadres of maternal care providers. The training lasts for approximately 4–6 h and is scheduled around the daily work of the staff. The curriculum includes theoretical and practical aspects of care, supported by pictorial flip-charts and MamaNatalie® birthing simulators, low-cost birthing simulators that can create normal and complex birthing scenarios. The birthing simulators are left in the facility after the training. The one-day training is followed by 8 weeks of in-service practice facilitated by clinical mentors. These mentors receive an additional half-day of training to guide the 8 subsequent weeks of peer practising using the MamaNatalie® birthing simulators. Mentors are supposed to organise and support these ‘low-dose, high-frequency’ weekly practice sessions which are anticipated to last 15–20 min each.

The curriculum of the main training includes (1) communication between staff and the mother, (2) preparation for a clean and safe delivery, (3) measuring blood loss, (4) checking the uterus tone postpartum, (5) routine care for mother and baby, (6) Active Management of the Third Stage of Labour (AMTSL), (7) cord cutting, (8) placenta delivery, (9) checking the placenta for completeness, (10) decision making and organisation of referral in case of complications, (11) assessment of tears as a cause of bleeding and (12) bimanual compression of the uterus. Much emphasis is placed on highlighting the provision of good care for the mother and the baby, thus the training links to the Helping Babies Breathe training. The curriculum does not cover more advanced emergency obstetric care interventions like manual removal of the placenta, balloon tamponade or surgical care.

### Inclusion of participants

The study includes 20 districts in Tanzania and 18 districts in Uganda as ‘clusters’, each with the main hospital and two large high-case-load health centres included, all of which offer at least some basic emergency obstetric care functions such as management of PPH or pre-eclampsia. Each cluster ought to have had a combined minimum of at least 3000 deliveries in the 9 months previous to the study. This inclusion criterion was important so that we could be confident that the included facilities cater for most of the complications with referral to facilities outside the study area being uncommon. In order to avoid contamination, districts which were receiving training in safe motherhood or emergency obstetric care by other development partners were excluded. Within the included facilities and districts we recruited all pregnant women who had a complication. Using the World Health Organisation (WHO)-promoted disease and management-based near-miss form, we then selected those women who fulfilled the near-miss criteria.

In Tanzania the four study regions were purposefully selected by the Ministry of Health, Community Development, Gender, Elders and Children on the basis of being areas with high maternal mortality in the country. Of the four regions selected, we excluded two districts in southern Tanzania as well as two districts in the north-eastern region as they did not have the expected health care infrastructure of a hospital and two larger health centres. We selected all 20 public and faith-based hospitals except the university hospital in Mwanza, which serves as a zonal referral facility. In each of the districts we included the largest health centres, regardless of whether they were managed publicly or faith-based. We include 61 facilities in total from the four regions: 15 facilities from Lindi, 15 facilities from Mtwara, 16 facilities from Mwanza and 15 facilities from Simiyu. Among the 61 facilities, six were faith-based facilities.

In Uganda, we included 18 districts from the Central and Eastern regions of the country based on recommendations of the Ministry of Health. We included seven districts from the Eastern region and 11 from the Central region. A total of 22 hospitals, 16 health centre IVs (typically providing inpatient and outpatient services including basic emergency obstetric care) and 5 high-volume health centre IIIs (typically providing delivery care and some emergency obstetric care functions) were included. Among the 43 facilities, 8 were faith-based and 1 was managed by a non-government organisation.

### Evaluation methodology

The evaluation is based on the primary outcome of severe maternal morbidity and several secondary outcomes which include preventive actions and case management of PPH; knowledge, skills and confidence in preventing and managing PPH; and facility readiness for prevention and care of PPH (Table 1). The evaluation uses the framework proposed by Kirkpatrick to assess training interventions along the pathway of impact from reaction, learning, behaviour and results [31]. We also assess the facility readiness — the capacity of the facility in terms of trained staff, equipment and supplies — to care for women with PPH, as this is likely to have an important impact on the application of the new knowledge and skills [32, 33].

**Table 1:** Primary and secondary study outcomes

Primary outcome indicators	
Severe maternal morbidity	• PPH near-miss cases among all women who delivered in the facility • PPH near-miss cases among women who suffered PPH during health facility delivery • Near-miss cases among all women who delivered in the facility
Secondary outcome indicators	
Mortality (case fatality) in PPH near misses	• Deaths in PPH near-miss cases
Preventive actions and case management for PPH	• Proportion of women who received AMTSL of total women with PPH • Proportion of women with haemoglobin less than 70 g/L before discharge among those who suffered PPH • Proportion of women who received blood transfusion, intravenous oxytocin, balloon tamponade or a hysterectomy among those who suffered PPH • Proportion of women who need emergency transfer to a higher-level facility among those who suffered PPH in the health centre
Knowledge, skills and confidence in preventing and managing PPH	• Proportion of health care providers passing the knowledge test assessing preventive and emergency PPH care • Confidence score summing five questions about AMTSL, PPH, assessing advanced care, completeness of the placenta and bimanual uterine compression • Clinical skills score summing routine care including AMTSL, retained placenta and severe PPH • Team work
Facility readiness for prevention and care of PPH	• Proportion of facilities with AMTSL protocols and emergency tray available on day of visit • Proportion of facilities with at least a team of two midwives and one clinician on day of visit • Proportion of facilities with oxytocin and infusion set available in delivery room on day of visit • Stock-outs of oxytocin

The primary outcome of this study is severe maternal morbidity defined by the WHO disease and management-based near-miss criteria and referring to women while pregnant or within 42 days of termination of pregnancy, excluding accidental or incidental cases [34]. We modified the original WHO definition criteria as proposed by Nelissen et al. [35] and supported by a recent study which indicated that restricting near misses to cases of organ dysfunctions excludes many severe cases, even in high-income settings like the Netherlands [36]. Thus we defined a near-miss case as a woman experiencing either eclampsia, sepsis or uterus rupture OR any organ dysfunction using the standard near-miss definitions OR having received blood transfusion or a hysterectomy. The indicators are constructed using the total number of deliveries reported by the facilities (indicators one and two). For indicator three ‘PPH-related near-miss cases among women who suffered PPH in health facility delivery’ we use the number of women experiencing PPH in the facility as the denominator. We measure case fatality in PPH near misses as a secondary outcome. Also, several secondary outcomes are selected to reflect knowledge and management along the pathway of implementation including equipment availability and supplies.

## Data collection method

### Severe maternal morbidity and preventive actions and case management for PPH

We use the near-miss form as proposed by WHO to collect outcome data [34]. We include some background information, including use of induction/augmentation of labour, estimated blood loss and patient’s age and parity. We also added variables to capture the number of units of blood transfused and the haemoglobin at discharge for mothers who experienced haemorrhage or received a blood transfusion.

We trained two members of the maternity staff, midwives or clinicians, to be data collectors in each of the included health facilities. Their role is to abstract the relevant information each morning from the patient notes according to a standard protocol. Data collectors visit antenatal, labour and postnatal wards, the female general and female wards and the laboratory as well as attending the morning report session to ensure that all cases of near misses and female deaths are reviewed. All cases which could potentially qualify as a near miss are to be captured by the data collectors, including every case of haemorrhage regardless of the severity. Data are abstracted from multiple sources including admission, discharge and birth registries as well as case notes. The data collectors use a paper-based form and then enter the anonymised data immediately at discharge or death into the tablet-based application (Lenovo A3500-F). The application uses ranges and completeness checks to ensure data quality and completeness. The data collectors upload the data from the tablet to the cloud on a bi-weekly basis.

Training for data collectors is initially provided during a one-and-a-half day training session which includes (1) the concept of near miss, (2) collecting data using the near-miss forms and (3) the tablet application, procedure for uploading data, data safety and handling of missing data. We put an emphasis on the definition of PPH and its recognition in order to improve recording of PPH. Data collectors are retrained in a one-day refresher session after 5 months of data collection by reviewing forms with the data collectors and discussing their experiences and problems encountered.

To ensure compliance with the data collection protocol, project staff will conduct monthly visits at the beginning, then 3-monthly supervision visits as well as monthly telephone calls to the data collectors. Registers and files are reviewed together at in-person meetings to check for missed cases or missing information. Data collectors in the health centres are linked to the nearest study hospital for support, and WhatsApp groups are established for exchange on technical issues between data collectors and research team.

Data on the denominator — the total number of deliveries in the facilities — is collected through the monthly telephone call and verified during supervision visits. Near-miss data are checked once a month against the reported number of severe events to ensure all data has been uploaded successfully. In addition, we have an external team perform a quality control for completeness by randomly selecting a sample of one third of the facilities. They then review the registers and case notes and extract the respective information to define cases for the last month to compare results with the information submitted by the facility-based data collectors.

### Knowledge, skills and confidence in preventing and managing PPH

We will evaluate the knowledge, skills and confidence of health care providers before and after the competency training on the same day of the training using the standard Jhpiego tool. The confidence assessment involves self-rating on performance of AMTSL and PPH management.

### Facility readiness for prevention and care of PPH

We will evaluate the readiness of facilities to provide care for PPH complications using a health facility assessment tool adapted from tools used earlier in Tanzania and Uganda [25, 37]. We included items which indicate the availability of PPH services, including blood transfusion, human resources, drugs and supplies, protocols for preventive and curative action for PPH and emergency preparedness (emergency trays, referral readiness including transport and communication). Each facility involved in the study is visited twice: once at the start of the baseline period of data collection and again at the end of the 9-month intervention period to collect this data.

We established a scientific advisory committee to monitor the trial. Stopping rules were not agreed on, as the intervention was a single event and no harms were expected. Endline data collection is ongoing in Tanzania (February to October 2016) and is planned to start in Uganda in December 2016 and to run up to September 2017.

### Sample size

We estimate six cases of near miss per 100 deliveries of which two will be PPH-related. This cautious assumption is based on studies from Africa where the PPH-related near misses per 100 deliveries ranged from 2% in a rural referral hospital in Tanzania [35], to 5% in Zimbabwe and Zambia [38], and 9% at a tertiary hospital level in Nigeria [39]. This range is broadly supported by a recent systematic review of severe maternal morbidity in sub-Saharan Africa [6].

We assumed that the training would reduce severe morbidity (defined as near miss) by 25%. This estimate was chosen because similar training had an effect on neonatal mortality of a similar size [11]. Secondly, it has been shown that consistent use of AMTSL should reduce severe outcomes by half [40]. Other interventions included in the training, such as prompt identification of haemorrhage and immediate treatment with oxytocin, should further reduce severe outcomes [7]; thus a reduction of 25% was deemed to be possible.

We used the formula proposed by Hayes and Moulton for matched clusters [41] to calculate the number of clusters needed in order to obtain a power of 80% in our analysis. Considering the estimated coefficient of variation of near misses between clusters, we estimate that the intervention will reduce overall near misses and PPH near misses per 100 deliveries by 25% in the intervention compared to comparison clusters (districts). Anticipating 3000 deliveries in each cluster (hospital and health centres), the following formula was used:

$$ \mathrm{c}=2+{\left({\mathrm{z}}_{\alpha /2}+{\mathrm{z}}_{\beta}\right)}^2\;\mathrm{x}\;\left[{\mathrm{p}}_0\left(1-{\mathrm{p}}_0\right)/\mathrm{n}+{\mathrm{p}}_1\left(1-{\mathrm{p}}_1\right)/\mathrm{n}+{\mathrm{k}}^2\left({{\mathrm{p}}_0}^2+{{\mathrm{p}}_1}^2\right)\;\right]/{\left({\mathrm{p}}_0-{\mathrm{p}}_1\right)}^2 $$

where z_α_/2 + z_β_ = (1.96 + 0.84) assuming a power of 80% and 5% error.

Considering the prevalence of near-miss haemorrhage equal to 2% in the comparison group, and assuming a *k* (the coefficient of the variation of the proportion of near misses between clusters) value of 0.15 [42], we calculated that the required number of clusters required is equal to 10 per group.

- *p*_0_ = 0.02
- *p*_1_ = 0.015 (reduction of 25%)
- *n* = 3000 deliveries in a cluster
- *k* = 0.15

Considering instead the prevalence of overall near miss equal to 6% in the comparison group, the number of clusters required is equal to 8 per group.

- *p*_0_ = 0.06
- *p*_1_ = 0.045 (reduction of 25%)
- *n* = 3000 deliveries in a cluster
- *k* = 0.15

### Randomisation

We use unblinded matched-pair randomisation of districts (clusters), which is done separately in each country. Criteria for matching were health care organisation (number of hospitals and health centres), urban/rural district, geographical area and a balance in the outcome measurement ‘PPH-related near-miss cases among women who suffered PPH’ available from the baseline data collection. Randomisation is done using Stata by the trial statistician, who is otherwise not involved in the study implementation. Blinding of participants is not possible due to the nature of the intervention.

### Analytical methods

Statistical analysis will be performed using Stata version 13 (College Station, TX, USA). An analytical plan is prepared for review by the international scientific advisory board.

Descriptive analyses included frequencies for categorical variables, and mean and standard deviation or median and inter-quartile range for continuous variables will be performed. The main analysis will compare districts with their included hospitals and health centres allocated to the HMS BAB training with the comparison districts, which will only receive the training at the end of the study. The analysis will be conducted on an ‘intention-to-treat’ basis. We will conduct an interrupted time series analysis in order to compare the changes in the proportion of near misses before and after the intervention period between the two groups (intervention and comparison) at the cluster level. The analysis will use the difference-in-differences estimate comparing changes within each district.

In order to compare individual-level skills and competence of trained health care providers (using the pre-post training evaluation), we will use multilevel mixed-effects generalised linear models (Stata command *meglm*) with different family distributions according to the outcome distribution (Stata option *family*). Results will be presented as effect estimates with 95% confidence intervals and will be done separately for each country. Sub-group analyses for primary outcomes will be done for type of district (urban/rural). Interaction tests will be performed if relevant.

Comparing outcomes calculated at the cluster level is recommended if the cluster size is limited to 18–20 clusters. The restricted (matched) cluster-randomised allocation to implementation and comparison districts should ensure that both are comparable with respect to key factors. However, this assumption will be assessed by comparing key input characteristics such as human resources and major equipment and services available using chi-squared tests or, if significant, adding these characteristics to the regression models mentioned above to adjust for their confounding effect. The analysis of secondary endpoints of management practices will follow the same approach.

We will complement the analysis by carefully assessing differences between intervention and comparison districts throughout the implementation period. If relevant differences are observed, these factors influencing readiness will be included in the analysis of the effect of the intervention on the primary and secondary outcomes. In this case, Bonferroni and Šidák corrections for multiple comparisons will be performed.

### Data protection

Password-protected personalised tablets are used to collect the data. We only upload information on baseline information, complications and interventions without the name or other personal identifiable information to a secure cloud-based server. The data are cleaned by the trial team and stored on password-protected devices only. We have developed a data sharing and data protection guideline which should ensure the use of the cleaned and fully anonymous data for secondary data analysis by others at a later date.

### Dissemination plan

The implementing partners — national associations of gynaecology and obstetrics and midwives — will disseminate results at the national level including the districts and regions where the evaluation is implemented. Results and experiences will also be communicated in plain language for wider use in the community, within local governance structures and civil society. We will present findings at national and international conferences, and we will publish manuscripts in peer-reviewed journals.

## Discussion

The evaluation will estimate the effect of the HMS BAB training on maternal mortality and morbidity (near-miss) in a large two-country trial. The study will provide evidence on whether the training programme leads to improved maternal health outcomes. We will also be able to provide information on ’lessons learned’ in implementing the training including the 8-week in-service practice sessions which at medium scale can be used to guide future policy and investments regarding scale-up of training programmes for maternal health in the respective countries. Looking at the evaluation along the implementation pathway will allow us to propose some explanations in case we unexpectedly see no effect of the intervention.

### Limitations

The main limitation of the study is that primary and secondary outcome measurements are not collected by external staff. We decided to use facility staff for several reasons: firstly ,we estimated that the time spent on collecting data in facilities would take less than 1 hour per day, which makes it unnecessary to employ external staff (and this would greatly increase costs); secondly, we received advice from local collaborators and district managers that the acceptability of external data collectors might be limited over such a long period of time; and thirdly, even if external data collectors were used, they would still rely on case notes prepared by maternity staff who are, by the nature of such a training intervention, not independent.

In order to address this, we aim to standardise the data collection during the 6-month period prior to the randomisation to reduce the reporting bias potentially introduced by the lack of blinding. In addition, our supervision team will visit the facilities every 1–3 months to verify the data by checking the registries. We also include an external assessment by independent data collectors in a sample of facilities to investigate any recording bias and confirm data completeness.

Another limitation is that the HMS training itself might improve recognition of PPH complications and recording of management. Such an unexpected reverse effect was observed in the evaluation of the Helping Babies Breathe training in Tanzania [11].

Contamination is a major threat to our evaluation. Other training or skill-building approaches in the field of safe motherhood or emergency obstetric care provided by the ministries of health or development partners might contaminate the training approach in our intervention and comparison districts. We are not able to restrict these much-needed development initiatives, as this would be unethical, but we document this when it occurs so that we are able to adjust for it in our analysis. Contamination and overall health sector development has been described in several trials to improve maternal and child health as a potential reason why results were missed [43, 44].

Finally, it is common practice that health workers are transferred to other facilities. In response, facilities in intervention districts might lose trained staff while facilities in comparison districts might then have health workers with improved skills to manage PPH.

Recruitment of participants was ongoing at submission of the protocol.

## Additional file

Additional file 1: SPIRIT checklist. (DOC 133 kb)

## Abbreviations

AMTSL: Active Management of the Third Stage of Labour
HMS BAB: Helping Mothers Survive Bleeding after Birth
PPH: Postpartum haemorrhage
WHO: World Health Organisation
